# Long-Term Impact of COVID-19: A Systematic Review of the Literature and Meta-Analysis

**DOI:** 10.3390/biomedicines9080900

**Published:** 2021-07-27

**Authors:** Diana C. Sanchez-Ramirez, Kaylene Normand, Yang Zhaoyun, Rodrigo Torres-Castro

**Affiliations:** 1 Department of Respiratory Therapy, College of Rehabilitation Sciences, University of Manitoba, Winnipeg, MB R3E 0T6, Canada; normandk@myumanitoba.ca; 2 School of Nursing, Jilin University, Changchun 130012, China; yzy13553100125@163.com; 3 Department of Physical Therapy, Faculty of Medicine, University of Chile, Santiago 8320000, Chile; rodritorres@uchile.cl

**Keywords:** COVID-19, persistent symptoms, long-term effects, follow-up, lung function, respiratory symptoms, fatigue, functional capacity, quality of life

## Abstract

Background: The long-term impact of COVID-19 is still unknown. This study aimed to explore post COVID-19 effects on patients chest computed tomography (CT), lung function, respiratory symptoms, fatigue, functional capacity, health-related quality of life (HRQoL), and the ability to return to work beyond 3 months post infection. Methods: A systematic search was performed on PubMed, Web of Science, and Ovid MEDLINE on 22 May 2021, to identify studies that reported persistent effects of COVID-19 beyond 3 months follow-up. Data on the proportion of patients who had the outcome were collected and analyzed using a one-group meta-analysis. Results: Data were extracted from 24 articles that presented information on a total of 5323 adults, post-infection, between 3 to 6 months after symptom onset or hospital discharge. The pooled prevalence of CT abnormalities was 59% (95% CI 44–73, I^2^ = 96%), abnormal lung function was 39% (95% CI 24–55, I^2^ = 94%), fatigue was 38% (95% CI 27–49, I^2^ = 98%), dyspnea was 32% (95% CI 24–40, I^2^ = 98%), chest paint/tightness was 16% (95% CI 12–21, I^2^ = 94%), and cough was 13%, (95% CI 9–17, I^2^ = 94%). Decreased functional capacity and HRQoL were found in 36% (95% CI 22–49, I^2^ = 97%) and 52% (95% CI 33–71, I^2^ = 94%), respectively. On average, 8 out of 10 of the patients had returned to work or reported no work impairment. Conclusion: Post-COVID-19 patients may experience persistent respiratory symptoms, fatigue, decreased functional capacity and decreased quality of life up to 6 months after infection. Further studies are needed to establish the extent to which post-COVID-19 effects continue beyond 6 months, how they interact with each other, and to clarify their causes and their effective management.

## 1. Background

The novel coronavirus disease 2019 (COVID-19), caused by severe acute respiratory syndrome coronavirus 2 (SARS-CoV-2) infection, has spread rapidly worldwide reaching over 191 million confirmed cases, including 4.1 million deaths as of 21 July 2021 [1]. Although lung abnormalities are found even in asymptomatic patients with COVID-19 [2], most of the cases (81%) have no or mild pneumonia and can be managed at home. Severe (14%) and critical (5%) cases develop severe pneumonia and respiratory failure, which require in-hospital oxygen treatment or mechanical ventilation and are more likely to have long-term effects [3].

Evidence from literature on viral pneumonia indicates that many recovered patients have significant lung changes and are affected by acute respiratory distress syndrome (ARDS), which can negatively impact their lung, physical function and quality of life for months or years [4,5]. Previous studies conducted on patients with severe acute respiratory syndrome (SARS) caused by a SARS coronavirus that was identified in 2003 report long-term decreases in pulmonary function, including reduced lung volume measurement in 7.3% of the patients at 6 months [6]. The most common pulmonary function impairment identified at a 1 year time point was of the forced expiratory volume (FEV1) and diffusing lung capacity for carbon monoxide (DLCO) which were still abnormal in 18.2% and 52.7% of patients, respectively, at 24 months post-SARS [7,8]. Decreases in exercise capacity and health-related quality of life were also found in SARS patients, and only 78% of SARS survivors had returned to work within 24 months [7]. Similarly, signs of severe abnormal pulmonary function were reported in 54.2% of patients with influenza A (H1N1), 1 year post-discharge [9], 59% of the patients reported significant exertion dyspnea, 70% had returned to work, health-related quality of life was poorer than for sex- and age-matched general population groups [10]. Overall, evidence indicates that ARDS patients experience decreases in lung function, exercise endurance, levels of body function (muscle strength, walking capacity, and/or physical activity), and HRQoL, and lower return-to-work rate [11,12,13]. As COVID-19 has recently emerged, the long-term impact of the disease, defined as the signs and symptoms lasting beyond the period of active infection, is just becoming known.

In recent months, the scientific literature has used the term “Long COVID” to describe illness in people who have reported lasting effects of the infection or have had the usual symptoms for far longer than would be expected [14]. Abnormal chest computed tomography (CT) findings and impairment in lung function were found in COVID-19 patients 30 days after hospital discharge [15]. Persistent symptoms such as cough, dyspnea, and fatigue have commonly been reported in post COVID-19 patients between 30 and 56 days after hospital discharge [15,16,17,18,19]. Decreased functional capacity [17] and difficulties with the activities of daily living such as mobility and self-care [17,18,19] were also identified in patients for follow-ups of up to 8 weeks [20]. A study reports that 39% of patients perceived a decrease in their overall health [19] around 6 weeks after symptom onset. 61.5% of patients delayed their return to work at least 5 weeks from symptom onset, the primary reason for which was fatigue and weakness [16], and 38% were absent due to illness [19]. Evidence indicates that COVID-19 can have significant persistent effects on patient outcomes up to 12 weeks after the period of acute infection [21,22,23]. However, the long-term impact of COVID-19 on patient outcomes is still unknown. The aim of this study is to explore persistent effects of COVID-19 beyond 3 months follow-up on patient chest computed tomography (CT), lung function, respiratory symptoms, fatigue, functional capacity, quality of life, and the ability to return to work. The results of this study will help fill a significant knowledge gap about the lingering effects of COVID-19 on patient outcomes, which are valuable for tailoring rehabilitation programs to the needs of COVID-19 patients and for informing future health care planning and resource allocation.

## 2. Methods

### 2.1. Protocol and Registration

This systematic review of the literature was conducted using Preferred Reporting Items for Systematic Reviews and Meta-Analyses (PRISMA) [24]. It was registered in the International Prospective Register for Systematic Reviews (PROSPERO) (CRD42021256958).

### 2.2. Literature Search and Study Selection 

A systematic review was performed in the electronic bibliographic databases of PubMed, Web of Science and Ovid MEDLINE on 22 May 2021. The key words and the search strategy used were: (“COVID-19” OR “COVID19” OR “SARS-2” OR “Severe acute respiratory syndrome 2”) AND (“follow-up” OR “follow-up studies” OR “lingering symptoms” OR “persistent symptoms” OR “long-term symptoms”). The search was limited to full text articles published in English. In addition, the reference lists of the selected studies were checked to retrieve relevant publications that were not found with the computerized search.

A total of 24 publications met the inclusion criteria of: (1) reporting the effect of COVID-19 on one or more of the following outcomes in adult patients’ lung imaging, lung function, respiratory symptoms, fatigue, functional capacity, activity limitations, overall health, quality of life, or return to work; (2) the mean or median study follow-up was longer than 3 months; (3) the publication was not a meta-analysis or systematic review. The articles selected were appraised by two review authors (RT and YZ) using the tool recommended by the National Heart, Lung, and Blood Institute (NHLBI) for the quality assessment of observational cohort and cross-sectional studies [25]. 

**Figure 1:**
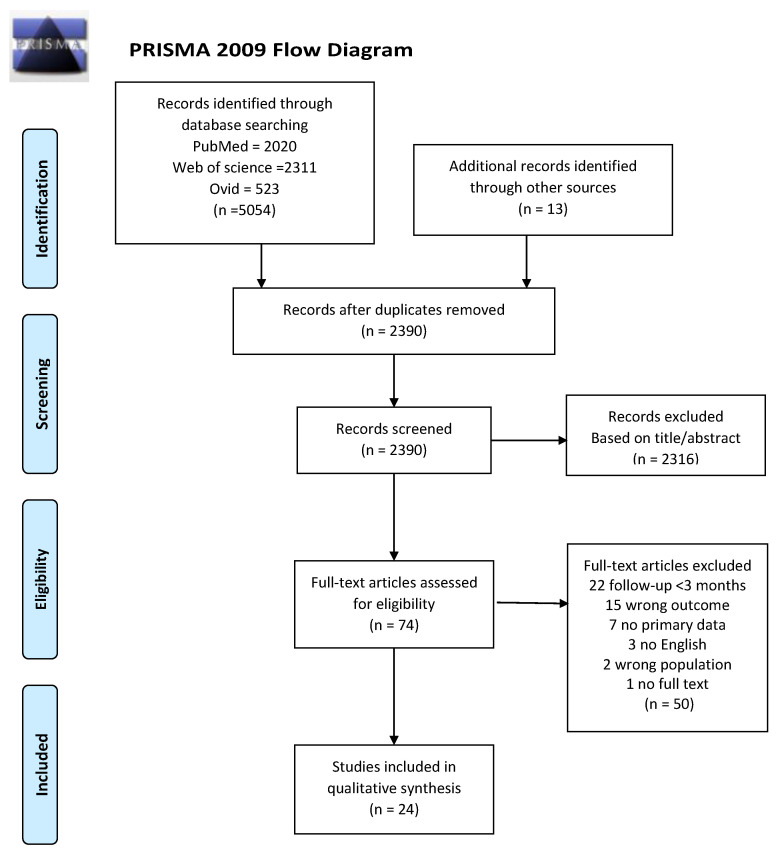
Diagram flow of studies screened and included in the review and meta-analysis.

The systematic search retrieved 5054 references and 13 references were identified from other sources such as automatic email alerts on COVID-related databases, reference lists of identified studies, links shared on social media, suggested by a researcher, etc. After removing duplicates, two researchers (DS and KN) screened 2390 titles and abstracts and read in full the text of 74 articles. Both researchers independently reviewed the articles and selected 24 publications that met the inclusion criteria (Figure 1). Primary reasons for exclusion of the studies included; (1) the study did not present the effect of COVID-19 on any of the following outcomes: lung imaging, lung function, respiratory symptoms, fatigue, functional capacity, activity limitations, overall health, quality of life, and/or the ability to return to work; (2) the study was completed in children or in a group of post COVID-19 adults with a specific characteristic or disease (e.g., among patients with diabetes, or kidney problems); (3) the follow-up was less than 3 months; and (4) the article did not present primary data (e.g., protocol, opinion, etc.).

### 2.3. Data Extraction and Synthesis 

Information from the 24 articles is synthesized in Table 1 which presents (1) author(s)’ name, (2) country of the study, (3) study design, (4) follow-up time, (5) description of the population studied and, (6) disease severity at baseline. Prevalence of persistent COVID-19 effects beyond 3 months of follow-up on chest CT scan, lung function, respiratory symptoms, fatigue, functional capacity, quality of life, and return to work of patients was extracted and is compiled in Table 2.

Review Manager (RevMan; version 5.3; Copenhagen, Denmark) software was used to pool the individually included studies. The analysis is presented as prevalence based on the presence of the outcome using one-group meta-analysis, and random-effects methods. To investigate heterogeneity between studies, the authors used the I^2^ index which describes the percentage of variation across the studies in the pooled analysis that is due to inconsistency rather than by chance. The I^2^ statistic was used to present between-study heterogeneity, where I^2^ ≤ 30%, between 30% and 50%, between 50% and 75%, and ≥75% were considered to indicate low, moderate, substantial, and considerable heterogeneity, respectively [26].

## 3. Results

This review identified a total of 24 articles that explore persistent effects of COVID-19 beyond 3 months of follow-up on patients lung imaging, lung function, respiratory symptoms, fatigue, functional capacity, HRQoL, and/or the ability to return to work [27,28,29,30,31,32,33,34,35,36,37,38,39,40,41,42,43,44,45,46,47,48,49,50]. The studies were completed in China (7), Canada (3), France (2), Norway (2), Italy (3), USA (2), Switzerland (1), Austria (1), Iran (1), the Netherlands (1), and the UK (1). Persistent outcomes were reported in 5323 post COVID-19 adult patients at an average of 4 (range 3–6) months of follow-up after symptom onset or hospital discharge. Mean age was 55.2 ± 8.1 years, and 56% of the patients studied were male. Severe illness due to COVID-19, defined as the presence of pneumonia, serious or critical illness, need for hospitalization, ICU care, use of supplemental oxygen, etc., was reported in approximately 36% of the cohort at baseline. Further description of the studies is presented in Table 1. Two of the Canadian studies [35,37] presented information on the same population. Data from Wong et al. [35] were preferred when the same outcomes are reported in both studies due to longer follow-up and a larger number of patients studied; otherwise, the unique results presented in each study were included in this review.

The NHLBI’ Quality Assessment Tool for Observational Cohort and Cross-Sectional Studies was used to evaluate the quality of the 24 studies included in the review. Nineteen studies were rated as “fair” and five rated as “poor” (Supplementary Table S1).

### 3.1. Lung Imaging

Thirteen articles report the results of chest CT scans in post COVID-19 patients at a mean of 3.8 (SD 1.3) months follow-up [28,29,30,31,32,33,34,36,37,40,41,43,45] (Supplementary Table S2 and Figure S1). Persistent chest CT abnormalities attributable to COVID-19 were identified in 59% of the patients (95% CI 44–73, I^2^ = 96%) (Table 2). The most frequent CT abnormality was ground glass opacity (GGO) in 39% of the patients (95% CI 26–52, I^2^ = 97%), followed by interstitial thickening or interlobular septal thickening in 33% (95% CI 13–52, I^2^ = 98%), parenchymal band or fibrous stripe in 31% (95% CI 17–44, I^2^ = 95%), bronchovascular bundle distortion or bronchiectasis in 26% (95% CI 9–43, I^2^ = 97%), thickening or adjacent pleura in 11% (95% CI 2–20, I^2^ = 94%), and consolidations in 6% (95% CI 2–11, I^2^ = 89%). One study reports crazy pavement in 5% of participants a mean of 93 days after discharge from hospital. Residual lung lesions at follow-up were associated with disease severity during the infectious period in some studies [30,33,34,41,45] (Supplementary Table S2 and Figure S1).

### 3.2. Lung Function

Thirteen articles explored the effect of COVID-19 on lung function at follow-up [27,28,29,30,31,32,36,37,40,41,43,45,46] (Supplementary Table S3 and Figure S2). Abnormal pulmonary function tests (PFT) were identified in 39% (95% CI 24–55, I^2^ = 94%) of the patients (Table 2). Impaired diffusion lung capacity was the most common finding in 31% (95% CI 24–38, I^2^ = 89%) of the patients assessed, followed by restrictive pattern in 12% (95% CI 8–17, I^2^ = 82%), and obstructive pattern in 8% (95% CI 6–9, I^2^ = 7%). DLCO was lower in hospitalized vs. non-hospitalized patients, however, abnormal values were observed in both groups [46]. Mean DLCO was significantly lower in moderate-to-critical disease patients compared to referred mild disease patients who had normal mean DLCO [34,45]. No impact of disease severity in lung function are reported in other studies [30,41].

### 3.3. Fatigue and Respiratory Symptoms

Fifteen studies explored persistent post COVID-19 fatigue [29,31,32,35,36,39,40,41,43,44,45,46,47,49,50] and eighteen studies [27,28,29,30,31,32,33,35,36,40,41,42,43,44,46,47,49,50] report on one or more persistent respiratory symptoms at follow-up (Supplementary Table S4 and Figure S3). Pooled analysis indicates that fatigue was reported by 38% of the patients (95% CI 27–49, I^2^ = 98%) (Table 2), dyspnea by 32% (95% CI 24–40, I^2^ = 9%), chest pain or tightness by 16% (95% CI 12–21, I^2^ = 94%), cough by 13%, (95% CI 9–17, I^2^ = 94%), sputum by 12% (95% CI 3–21, I^2^ = 94%), and sore throat by 4% (95% CI 2–7, I^2^ = 66%). One study reports a higher prevalence of fatigue in patients with severe COVID-19 [29], and another found a lower prevalence of dyspnea (mMRC) in patients with moderate-to-critical illness compared with patients with mild disease [45]. However, disease severity is not significantly associated with the prevalence of fatigue or respiratory symptoms at follow-up in other studies [30,35,41,42,44].

### 3.4. Functional Capacity, HRQoL, and Return to Work/No Work Impairment

Decreased functional capacity was identified in an average of 36% (95% CI 22–49, I^2^ = 97%) of the patients at a mean of 4.2 (SD 1.3) months follow-up using data from five studies which presented a proportion of decreased functionality assessed with the work productivity and impairment questionnaire (WPAI) [49] and with performance-based tests (Table 2) including the short physical performance battery (SPPB) [27,48], the one minute sit to stand test (1-MSTST) [48], the 2 min walking test [27,48], and the 6 minute walking test (6MWT) [29,45] (Supplementary Table S5 and Figure S4). Cao et al. also stated that performance in the 6MWT was significantly lower in post COVID-19 patients than in health controls but did not report the proportion of patients under the threshold [43]. Lower performance in the 6MWT was reported in patients with severe/critical COVID-19 compared to patients with mild/moderate disease at baseline [29,34,47,49], however, another study reports no difference between groups [30]. 

Four studies report decreased HRQoL in an average of 52% (95% 33–71, I^2^ = 94%) of the participants at a mean of 4.5 (SD 1.7) months follow-up (Table 2) assessed with the Nijmegen Clinical Screening Instrument (NCSI) [45], a sliding scale ranging from 0 to 100 (best) [39], and using the 5 level EuroQoL 5 Dimensions (EQ-5D-5L) [35, 38] (Supplementary Table S5). Patients who experienced severe COVID-19 had larger declines in HRQoL (EQ-5D-5L) [29,30,38]. Conversely, patients with mild disease reported a significatively worse health status on most subscales of the SF-36, and on the subdomains of the NCSI, compared with moderate-to-critical disease patients [45]. No significant correlation between HRQoL or level of physical activity and disease severity at baseline is reported in other studies [44,47].

Results from four studies indicate that on average 80% of patients had returned to their previous work or reported no work impairments at a mean of 3.4 (SD 0.4) months follow-up [31,40,44,49] (Supplementary Table S5). A large percentage of ward hospitalized patients (75.6%) returned to work compared to ICU patients (46.7%), and more work impairment was reported in hospitalized patients (58.3%) compared to those not hospitalized (35.0%), however, the differences were not statistically significant [44,49]. Pooled analysis was not completed as one study identified that all the participants had returned to work.

## 4. Discussion

Results of this literature review and meta-analysis indicate that post-COVID-19 patients may experience persistent chest CT abnormalities, decreased lung function, persistent fatigue, and respiratory symptoms, decreased functional capacity, and decreased quality of life up to 6 months after symptom onset or hospital discharge. On average, 8 out of 10 patients had returned to work or reported no work impairment at around 3 months of follow-up.

Prevalence of chest CT abnormalities and decreased pulmonary function post-COVID-19 aligned with evidence from previous studies, which report abnormal outcomes in patients with viral pneumonias up to 64 months after illness onset [51,52]. Persistent lung abnormalities are significantly associated with disease severity at baseline in various studies [30,33,34,41,45]. However, high prevalence of chest CT abnormalities was not well reflected on PFT and only weak to moderate correlations were found [31,32]. Presence of GGO, commonly identified at follow-up [28,29,30,31,32,33,34,36,37,40,41,43,45], can be a manifestation of a wide variety of clinical features including focal interstitial fibrosis, and inflammation [53]. Residual pulmonary parenchymal abnormalities were correlated with lower DLCO [45], and pulmonary function abnormalities occurred frequently in patients with fibrotic-like changes [28]. Impaired DLCO reported in the PFT [27,28,29,30,31,32,36,37,40,41,45] can be present in interstitial lung diseases or pulmonary fibrosis, which are clinical conditions where the surface area of the alveolar membrane is reduced [54]. DLCO values represent the ability of the lung to transfer gas from the inhaled air into the blood stream and can be used as a marker of the extent of lung damage [55]. Impaired DLCO may be attributed to abnormalities in the distal airways (such as constrictive bronchiolitis with air trapping and secondary reflex vasoconstriction), or to primary vascular disease (that may induce secondary airway disease) [34]. Abnormal DLCO and GGO on chest CT were associated with duration of oxygen supplementation [37].

High prevalence of cough, dyspnea, and fatigue were reported in post COVID-19 patients. Cough and dyspnea can be indicators of impaired lung function [54] and may also affect the execution of an adequate PFT [56]. Dyspnea scores were found to correlate with PFT measurements in COVID-19 patients [31]. Presence of dyspnea is attributed to abnormalities on lung CT scan in 44 of 78 patients (45%), including fibrotic lesions in 18 of 78 cases (23%) and to hyperventilation functional breathing test confirmed dysfunctional breathing in 14 of 78 patients (17%) [36]. Although some evidence supports the theory that breathing problems in COVID-19 patients may be due to fibrotic changes in the lung, it is also suggested that post-viral autonomic dysfunction may contribute to the cause of this and other persistent symptoms [57]. Fatigue, the most prevalent symptom reported by post COVID-19 patients, was identified in one third of the patients and could likely persist for a longer time. A previous study reports that 40% of SARS survivors had chronic fatigue for a mean of 41 months after infection [58]. Post-COVID-19 fatigue is not associated with disease severity in the acute phase or with abnormal chest CT abnormalities [22]. Fatigue was reported together with muscle weakness in one of the studies [42], and with physical decline in another [29]. It is possible that fatigue may have been caused by muscle weakness, respiratory symptoms, and general deconditioning. It has also been suggested that the coronavirus can trigger post-viral fatigue syndromes [59]. More studies will be needed to clarify the cause of fatigue in post-COVID-19 patients and the best way to treat it.

Decreased functional capacity was found in one third of post COVID-19 patients at follow-up. CT abnormalities are not associated with functional performance [41], however, residual signs suggesting pulmonary fibrosis are associated with exercise-induced desaturation [45]. Sixteen percent of patients desaturated upon the 6MWT, desaturators had lower DLCO than non-desaturators [45]. Persistent symptoms, decreased pulmonary function, muscle weakness and physical deconditioning may have contributed to post COVID-19 decreased function and perception of worsened HRQoL. Despite the higher prevalence of multiple post-COVID-19 effects, a large percentage of the patients had returned to work 3 to 4 months after discharge [31,40,44]. Importantly, all studies that reported on this outcome used small sample sizes (ranging from 55 to 76 patients). Furthermore, in the studies that found that all or almost all patients had returned to work, the participants were relatively young (mean age 41.3 SD13.8 and 47.7 SD 15.4 years) and only a small percentage of them had a serious illness that required ICU care (Table 1) [31,40]. Although in Garrigues et al. the group studied was older (mean 63.2 SD 15.7), this outcome was reported only in 46.7% (56/120) of the participants, probably younger patients still active in the workforce. Therefore, a potential explanation for the lower return to work (68%) could be a higher percentage of patients with severe disease at baseline, since 20% of participants were in the ICU [44]. It is also possible that the socio-economic context and available resources may have an impact on the return-to-work process. In general, more studies with larger sample sizes are needed to determine the potential long-term impact of COVID-19 on patients ability to return to work, as well as the possible causes and ways to mitigate them.

Patients with severe COVID-19 disease had higher prevalence of chest CT abnormalities [30,33,34,41,45]. However, inconclusive results were found regarding disease severity and persistent fatigue, respiratory symptoms, pulmonary function, functional capacity, and HRQoL. Evidence suggests that severity of disease during the acute period of infection may have an impact on persistent COVID-19 outcomes. However, only a few studies report the outcomes in relation with disease severity and no direct association between disease severity and post COVID-19 outcomes was observed. Persistent symptoms are also reported in patients with non-severe disease. 

### 4.1. Implications and Considerations

This study helps fill a significant knowledge gap about the lingering effects of COVID-19 on patient outcomes between 3 to 6 months after infection, which is very important considering the large number of COVID-19 patients who will potentially need help managing the impact of the disease beyond the infectious period. Outcomes of this study may help to understand the prevalence of persistent post-COVID-19 effects and their potential causes, which may guide the development of rehabilitation programs tailored to the needs of COVID-19 patients. From a public health perspective, the results of this study may help inform future health care planning and resources allocation. 

### 4.2. Study Limitations

Some factors should be considered when interpreting these findings. First, there is no data available on outcomes studied before the patients contracted COVID-19, therefore, it is unclear whether the abnormalities may have existed before the diagnosis of COVID-19. Second, it is possible that the presence of pre-existent comorbidities may have affected the results of the studies. However, Wong et al. [35] report that there were no differences in reported outcomes between patients with and without pre-existing major comorbidities, suggesting that many of the identified deficits are likely ongoing consequences of COVID-19. Third, there is heterogeneity across the studies with respect to the selection of participants, the assessment of outcomes, and the definition of the follow-up period in almost all the studies which may influence the generalizability of the results of this study. Despite these limitations, the authors believe that the results of this study contribute to filling a significant knowledge gap about persistent COVID-19 symptoms between 3 and 6 months after acute infection.

## 5. Conclusions

The results indicate that post-COVID-19 patients may experience persistent respiratory symptoms, fatigue, decreased functional capacity, and decreased quality of life up to 6 months after infection and, on average, around 8 out of 10 of the patients had returned to work at around 3 months follow-up. Outcomes suggest that, in addition to disease severity and lung injury, other potential causes of persistent symptoms, such as post-viral autonomic dysfunction and fatigue syndrome should be assessed and considered when planning post-COVID-19 rehabilitation interventions. This study may guide the development of rehabilitation programs tailored to the needs of COVID-19 patients and inform future health care planning and resource allocation. Further studies are needed to establish the extent to which post-COVID-19 effects continue beyond 6 months, how they interact with each other, and to clarify their causes and their effective management.

## Figures and Tables

**Table 1:** Characteristics of the studies included (*n* = 24).

Author Year	Country	Study Design	Follow-Up Time	Post COVID-19 Participants			Disease Severity at Baseline ^¥^
				N	Age (Years)	Male (%)	Severe Cases *n* (%)
Abdallah, S. 2021 [46]	Canada	Cross-sectional	119.9 (SD 16.2) days after positive COVID-19 test for hospitalized patients and 129.8 (SD 16.5) for non-hospitalized patients	63	59.1 (SD 13.5) for hospitalized 42.4 (SD 12.9) days non-hospitalized	57%	25 (40%) hospitalized
Anastasio, F. 2021 [47]	Italy	Cross-sectional	135 (IQR 102–175) days after symptoms onset	379	56.0 (IQR 49–63)	46%	222 (58.6%) pneumonia
Baricich, A. 2021 [48]	Italy	Cross-sectional	124 (SD 17.5) days after hospital discharge	204	57.9 (SD 12.8)	60%	27 (13%) ICU
Bellan, M. 2021 [27]	Italy	Prospective	4 months after hospital discharge	238	61.0 (IQR 50–71)	60%	28 (12%) ICU
Cao, J. 2021 [43]	China	Prospective	3 months after hospital discharge	61	43.5 (SD 15.9)	54%	57 (94%) pneumonia severe pneumonia critical illness
Garrigues, E. 2020 [44]	France	Cross-sectional	110.9 (SD 11.1) days following admission	120	63.2 (SD 15.7)	63%	24 (20%) ICU
Guler, S. 2021 [34]	Switzerland	Prospective	128 (IQR 108–144) days from initial symptoms	113	60.3 (SD 12) Severe/critical COVID 52.9 (SD 11) Mild/moderate COVID-19	59%	66 (58.4%) severe/ critical disease
Han, X. 2021 [28]	China	Prospective	175 (SD 20) days after symptoms onset	114	54.0 (SD 12)	70%	114 (100%) severe disease
Huang, Ch. 2020 [29]	China	Ambi-directional	186 (175–199) days after symptom onset	1733	57.0 (IQR 47–65)	52%	1294 (75%) required supplemental oxygen (n = 1172) or HFNC, NIV, or IMV (n = 122)
Jacobson, K. 2021 [49]	USA	Cross-sectional	119.3 (SD 33) days after COVID-19 diagnosis	118	43.3 (SD 14.4)	53%	22 (18.6%) hospitalized
Lerum, T. 2020 [30]	Norway	Prospective	83 (73–90) days after hospital admission	103	59.0 (IQR 49–72)	52%	15 (14.6%) ICU
Liang, L. 2020 [31]	China	Prospective	3 months after hospital discharge	76	41.3 (SD 13.8)	28%	7 (9.2%) ICU
Logue, J. 2021 [39]	USA	Prospective	169 (SD 39.5) days after illness onset	177	48.0 (SD 15.2)	43%	16 (10%) hospitalized
Morin, L. 2021 [36]	France	Prospective	4 months after hospital or ICU discharge	478	60.9 (SD 16.1)	58%	142 (30%) ICU
* Shah, A. 2020 [37]	Canada	Prospective	11.7 weeks after symptoms onset	60	67.0 (IQR 54–74)	68%	46 (76.7%) required supplemental oxygen
Sonnweber, T. 2020 [32]	Austria	Prospective	103 (SD 21) days after diagnosis for second visit (100 days after onset)	145	57.0 (14)	55%	109 (75%) hospitalized
Sykes, D. 2021 [50]	UK	Cross-sectional	113 days (46–167) days post discharge	134	59.6 (SD 14.0)	66%	27 (20.1%) ICU
Tabatabaei, S. 2020 [33]	Iran	Retrospective	91 (SD 15.5) days after initial CT	52	50.2 (SD 13.1)	62%	NR
van den Borst, B. 2020 [45]	the Netherlands	Prospective	13.0 (SD 2.2) weeks after symptoms onset	124	59.0 (SD 14)	60%	46 (37%) severe/ critical disease
Walle-Hansen, M. 2021 [38]	Norway	Prospective	186 days after discharge	106	74.3 (range 60–96)	57%	27 (25.4%) ICU or intermediary ward
* Wong, A. 2020 [35]	Canada	Prospective	13 (IQR 11–14) weeks after symptoms onset	78	62.0 (SD 16)	64%	NR
Wu, Q. 2021 [41]	China	Prospective	6 months after discharge	54	47.0 (IQR 36–57)	59%	23 (42.5%) severe disease
Xiong, Q. 2021 [42]	China	Prospective	97 (95–102) days after hospital discharge	538	52.0 (SD 41–62)	46%	207 (38.5%) severe/ critical disease
Zhao, Y. 2020 [40]	China	Retrospective	3 months after symptom onset (64–93 days after discharged from hospitals)	55	47.7 (SD 15.49)	58%	4 (7.3%) severe disease

COVID-19 = coronavirus disease 2019; SARS-CoV-2 = severe acute respiratory syndrome coronavirus 2; SD = standard deviation; IQR = Interquartile range; CI = confidence interval. NR = Not reported. ICU = Intensive care unit. * Both studies reported on the same population: data from Wong et al. were preferred when reporting the same results due to longer follow-up and a larger number of patients studied; otherwise, the unique results presented in each study were included. HFNC = high-flow nasal for oxygen therapy; NIV = non-invasive ventilation; IMV = invasive mechanical ventilation. ¥ As described in the articles.

**Table 2:** Prevalence of persistent COVID-19 effects at 3 to 6 moths follow-up.

Outcome	No. of Studies	No. of Patients Analyzed	Mean (SD) Follow-Up Time (Months)	Pooled Prevalence (95% CI)	I^2^ %	*p*-Value
Chest CT [28,29,30,31,32,33,34,36,37,40,41,43,45]						
Chest CT abnormalities	10	987	3.9 (1.4)	59% (44–73%)	96	<0.001
GGO	13	1313	3.9 (1.3)	39% (26–52%)	97	<0.001
Interstitial thickening or interlobular septal thickening	7	885	3.8 (1.2)	33% (13–52%)	98	<0.001
Parenchymal band or fibrous stripe	6	815	3.8 (1.2)	31% (17–44%)	95	<0.001
Bronchovascular bundle distortion or bronchiectasis	5	437	4.5 (1.3)	26% (9–43%)	97	<0.001
Thickening or adjacent pleura	4	573	5.5 (0.8)	11% (2–20%)	94	<0.001
Consolidation	4	652	4.9 (1.3)	6% (2–11%)	89	<0.001
Crazy paving	1	55	3.0	5% (1–11%)	NA	NA
Pulmonary function [27,28,29,30,31,32,36,37,40,41,43,45,46]						
Pulmonary function abnormalities	6	439	3.5 (1.2)	39% (24–55%)	94	<0.001
Diffusion pattern	12	1490	4.0 (1.3)	31% (24–38%)	89	<0.001
Restrictive Pattern	8	921	3.8 (1.5)	12% (8–17%)	82	<0.001
Obstructive pattern	7	858	3.6 (1.3)	8% (6–9%)	7	<0.001
Fatigue [29,31,32,35,36,39,40,41,43,44,45,46,47,49,50] and respiratory symptoms [27,28,29,30,31,32,33,35,36,40,41,42,43,44,46,47,49,50]						
Fatigue	15	4118	4.0 (1.3)	38% (27–49%)	98	<0.001
Dyspnea	16	3526	4.0 (1.1)	32% (24–40%)	98	<0.001
Chest pain/Tightness	10	3728	3.9 (0.9)	16% (12–21%)	94	<0.001
Cough	14	2539	3.8 (0.9)	13% (9–17%)	94	<0.001
Sputum	4	783	3.8 (1.4)	12% (3–21%)	94	<0.001
Sore Throat	6	2554	4.4 (1.3)	4% (2–7%)	66	0.02
Functional capacity [27,29,45,48], Health-related quality of live (HRQoL) [35,38,39,45], and return to work/work impairment [31,40,44,49]						
* Decreased functional capacity	5	2364	4.2 (1.3)	36% (22–49%)	97	<0.001
Decreased HRQoL	4	474	4.4 (1.6)	52% (33–71%)	94	<0.001
¥ Return to work/No work impairment	4	259	3.4 (0.4)	80%	NA	NA

COVID-19 = coronavirus disease 2019. CT = Computed tomography. Restrictive pattern (VCMax %predicted < 80% or VCmax < LLN OR FVC % predicted < 70–80% or FVC<LLN OR TLC z-score < −1.64 or TLC % predicted <80%). Obstructive pattern (FEV1/FVC < 70% OR FEV1/VCmax < LLN%). Diffusion impairment (DLCO < 80% predicted OR DLCO < LLN). * Data on performance-based tests. SD = standard deviation. NA = not applicable. ¥ Mean instead of pooling is presented due to one study in that group reporting the outcome in all the population studied. The I2 statistic was used to present between-study heterogeneity, where I^2^ ≤ 30%, between 30% and 50%, between 50% and 75%, and ≥ 75% were considered to indicate low, moderate, substantial, and considerable heterogeneity, respectively.

## Supplementary Materials

The following are available online at https://www.mdpi.com/article/10.3390/biomedicines9080900/s1, Table S1: Quality Assessment Tool for Observational Cohort and Cross-Sectional Studies, Table S2: Prevalence of chest CT abnormalities ≥3 months post COVID-19, Table S3: Prevalence of pulmonary function abnormalities ≥3 months post COVID-19, Table S4: Prevalence of fatigue and respiratory symptoms ≥3 months post COVID-19, Table S5: Prevalence of decreases functional capacity and health-related quality of life, and return to work ≥3 post COVID-19, Figure S1: Forest plots for follow-up chest CT, Figure S2: Forest plots for follow-up pulmonary function, Figure S3: Forest plots for follow-up respiratory symptoms, Figure S4: Functional capacity and Health-related quality of life.
