# Supplementary material for: Long-Term Impact of COVID-19: A Systematic Review of the Literature and Meta-Analysis
Source: Biomedicines. 2021 Jul 27;9(8):900. doi: 10.3390/biomedicines9080900 (PMC8389585; doi:10.3390/biomedicines9080900)

Supplementary material

Table S1. Quality Assessment Tool for Observational Cohort and Cross-Sectional Studies

Author	Year	1	2	3	4	5	6	7	8	9	10	11	12	13	14	Total Score	Quality Rating
Abdallah, S. et al. [46]	2021	Y	Y	Y	Y	N	N	Y	Y	Y	N	N	N	Y	N	8/14 (57%)	Fair
Anastasio, F. et al. [47]	2021	Y	Y	Y	Y	N	N	Y	Y	Y	N	Y	N	Y	N	9/14 (64%)	Fair
Baricich, A. et al. [48]	2021	Y	Y	N	Y	N	N	Y	N	N	N	Y	N	Y	Y	7/14 (50%)	Fair
Bellan, M. et al. [27]	2021	Y	Y	N	Y	N	Y	Y	N	N	N	N	N	N	Y	6/14 (43%)	Poor
Cao, J. et al. [43]	2021	N	Y	Y	Y	N	N	Y	Y	Y	Y	Y	N	N	Y	9/14 (64%)	Fair
Garrigues, E. et al. [44]	2020	N	Y	N	Y	N	N	Y	Y	Y	N	N	N	N	N	5/14 (36%)	Poor
Guler, S. et al [34]	2021	Y	Y	Y	Y	N	N	Y	Y	Y	N	N	N	Y	Y	9/14 (64%)	Fair
Han, X et al. [28]	2021	Y	Y	Y	Y	N	N	Y	N	N	N	Y	N	Y	N	7/14 (50%)	Fair
Huang, C. et al. [29]	2021	Y	Y	Y	Y	N	N	Y	Y	Y	N	Y	NR	Y	Y	10/14 (71%)	Fair
Jacobson, K. et al [49]	2021	Y	Y	NR	N	N	N	Y	Y	Y	N	N	N	Y	N	6/14 (43%)	Poor
Lerum, T. et al [30]	2020	Y	Y	NR	Y	N	N	Y	Y	Y	Y	Y	N	NR	Y	9/14 (64%)	Fair
Liang, L. et al. [31]	2020	Y	Y	Y	Y	N	N	Y	N	Y	N	Y	N	Y	Y	9/14 (64%)	Fair
Logue, J. et al. [39]	2021	N	Y	NR	Y	N	N	Y	Y	Y	N	N	N	N	N	5/14 (36%)	Poor
Morin, L. et al. [36]	2021	Y	Y	Y	Y	N	N	Y	Y	Y	N	Y	N	N	N	8/14 (57%)	Fair
Shah, A. et al. [37]	2020	Y	Y	Y	Y	N	N	Y	N	Y	N	N	N	Y	Y	8/14 (57%)	Fair
Sonnweber, T. et al. [32]	2020	Y	Y	Y	Y	N	N	Y	Y	Y	N	N	N	Y	Y	9/14 (64%)	Fair
Sykes, D. et al. [50]	2021	Y	Y	N	Y	N	N	Y	Y	Y	N	N	N	Y	N	7/14 (50%)	Fair
Tabatabaei, S. et al. [33]	2020	Y	Y	NR	Y	N	N	Y	N	Y	N	Y	Y	NR	N	7/14 (50%)	Fair
Van den Borst, B. et al. [45]	2020	Y	Y	Y	Y	N	N	Y	Y	Y	N	Y	N	Y	N	8/14 (57%)	Fair
Walle-Hansen, M et al. [38]	2021	Y	Y	N	Y	N	N	Y	N	Y	N	Y	N	Y	N	7/14 (50%)	Fair
Wong, A. et al. [35]	2020	Y	Y	Y	Y	N	N	Y	N	Y	N	N	N	Y	N	7/14 (50%)	Fair
Wu, Q. et. [41]	2021	Y	Y	N	Y	N	N	Y	Y	Y	N	Y	N	Y	N	8/14 (57%)	Fair
Xiong, Q. et al. [42]	2021	Y	Y	Y	Y	N	N	Y	N	Y	N	N	N	N	N	6/14 (43%)	Poor
Zhao, Y. et al. [40]	2020	N	Y	Y	Y	N	N	Y	N	Y	N	Y	N	Y	Y	8/14 (57%)	Fair

Quality of included studies was assessed using the National Institutes of Health (NIH) Quality Assessment tool for Observational Cohort and Cross-Sectional Studies (https://www.nhlbi.nih.gov/health-pro/guidelines/in-develop/cardiovascular-risk-reduction/tools/cohort). **1**. Was the research question or objective in this paper clearly stated? **2**. Was the study population clearly specified and defined? **3**. Was the participation rate of eligible persons at least 50%? **4**. Were all the subjects selected or recruited from the same or similar populations (including the same time period)? Were inclusion and exclusion criteria for being in the study prespecified and applied uniformly to all participants? **5**. Was a sample size justification, power description, or variance and effect estimates provided? **6**. For the analyses in this paper, were the exposure(s) of interest measured prior to the outcome(s) being measured? **7**. Was the timeframe sufficient so that one could reasonably expect to see an association between exposure and outcome if it existed? **8**. For exposures that can vary in amount or level, did the study examine different levels of the exposure as related to the outcome (e.g., categories of exposure, or exposure measured as continuous variable)? **9**. Were the exposure measures (independent variables) clearly defined, valid, reliable, and implemented consistently across all study participants? **10**. Was the exposure(s) assessed more than once over time? **11**. Were the outcome measures (dependent variables) clearly defined, valid, reliable, and implemented consistently across all study participants? **12**. Were the outcome assessors blinded to the exposure status of participants? **13**. Was loss to follow-up after baseline 20% or less? **14**. Were key potential confounding variables measured and adjusted statistically for their impact on the relationship between exposure(s) and outcome(s)? **Total Score,** number of yes; **NA**, not applicable; **NR**, not reported, **N**, not present; **Y**, present

Quality Rating: Poor <50%, Fair 50-75%, Good ≥75%

Table S2. Prevalence of chest CT abnormalities ≥3 months post COVID-19 (number of studies=13)

Author	N	CT abnormalities % (n)	Parenchymal band or fibrous stripe % (n)	GGO % (n)	Consolidation % (n)	Interstitial thickening or interlobular septal thickening % (n)	Bronchovascular bundle distortion or bronchiectasis % (n)	Thickening or adjacent pleura % (n)	Pleural effusion % (n)	crazy paving % (n)
**Cao, Jie et al., 2021 [43]**	61	54% (33)	NR	15% (9)	NR	33% (20)	NR	NR	NR	NR
**Guler, Sabine et al., 2021 [34]**	52	NR	52% (27)	58% (30)	29% (15)	2% (1)	38% (20)	2% (1)	NR	NR
**Han, Xiaoyu et al., 2021 [28]**	114	78% (89)	NR	21% (24)	3% (3)	NR	24% (27)	32% (37)	NR	NR
**Huang Chaolin et al., 2020 [29]**	353	53% (186)	16% (56)	45% (158)	1% (4)	1% (3)	NR	4% (15)	NR	NR
**Lerum, Tøri V. et al., 2020 [30]**	103	NR	18% (19)	23% (24)	NR	NR	NR	NR	NR	NR
**Liang, Limei et al., 2020 [31]**	21	24% (5)	NR	24% (5)	NR	NR	NR	NR	NR	NR
**Morin, Luc et al., 2021 [36]**	171	NR	19% (33)	42% (72)	NR	19% (33)	NR	NR	NR	NR
**Shah, Aditi S. et al., 2020 [37]**	60	88% (53)	NR	83% (50)	NR	65% (39)	NR	NR	NR	NR
**Sonnweber, Thomas et al., 2020 [32]**	133	63% (84)	NR	39% (52)	7% (9)	51% (68)	6% (8)	NR	NR	NR
**Tabatabaei, Seyed M. H. et al., 2020 [33]**	52	42% (22)	19% (10)	37% (19)	NR	NR	NR	NR	NR	NR
**van den Borst, B et al. 2020 [45]**	84	90% (76)	64% (54)	87% (73)	NR	NR	61% (51)	NR	NR	NR
**Wu, Qian et al., 2021 [41]**	54	20% (11)	NR	19% (10)	NR	NR	2% (1)	9% (5)	NR	NR

**Zhao, Yu-miao et al., 2020 [40]**	55	71% (39)	NR	13% (7)	NR	27% (15)	NR	NR	NR	5% (3)

COVID-19= coronavirus disease 2019. n=population of the study. CT= Computed tomography. NR= not reported or reported in a different format. GGO=ground glass opacities.

Table S3. Prevalence of pulmonary function abnormalities ≥3 months post COVID-19 (number of studies=13)

Author	N	PFT abnormalities % (n)	Restrictive Pattern % (n)	Obstructive pattern % (n)	Diffusion pattern % (n)
**Abdallah, Sara et al., 2021 [46]**	63	NR	11% (7)	11% (7)	46% (29)
**Bellan, Mattia et al., 2021 [27]**	219	NR	NR	NR	52% (113)
**Cao, Jie et al., 2021 [43]**	61	10% (6)	NR	NR	NR
**Han, Xaoyu et al., 2021 [28]**	104	NR	NR	NR	26% (27)
**Huang Chaolin et al., 2020 [29]**	349	NR	16% (56)	6% (22)	33% (114)
**Lerum, Tøri V. et al., 2020 [30]**	103	NR	7% (7)	NR	23% (24)
**Liang, Limei et al., 2020 [31]**	76	42% (32)	NR	7% (5)	20% (15)
***Morin, Luc et al., 2021 [36]**	152	NR	NR	NR	22% (33)
**Shah, Aditi S. et al., 2020 [37]**	60	58% (35)	23% (14)	12% (7)	52% (31)
**Sonnweber, Thomas et al., 2020 [32]**	113	36% (48)	26% (29)	8% (11)	21% (28)
**van den Borst, B et al. 2020 [45]**	124	NR	6% (8)	11% (13)	34% (41)
**Wu, Qian et al., 2021 [41]**	54	41% (22)	7% (4)	NR	31% (17)
**Zhao, Yu-miao et al., 2020 [40]**	55	25% (14)	7% (4)	11% (6)	16% (9)

PFT= Pulmonary function test. COVID-19= coronavirus disease 2019. N=population of the study. NR= not reported or reported in a different format. Restrictive pattern (VCMax %predicted<80% or VCmax<LLN OR FVC % predicted<80% or FVC<LLN OR TLC z-score<-1.64 or TLC % predicted<80%). Obstructive pattern (FEV1/FVC<70% OR FEV1/VCmax<LLN%). Diffusion impairment (DLCO<80% predicted OR DLCO<LLN). *DLCO <70%

Table S4. Prevalence of fatigue and respiratory symptoms ≥3 months post COVID-19 (number of studies=15 fatigue-16 respiratory symptoms)

Author	N	Fatigue % (n)	Dyspnea % (n)	Cough % (n)	Chest pain/ Tightness % (n)	Sore Throat % (n)	Sputum % (n)
**Abdallah, Sara et al., 2021 [46]**	63	71% (45)	60% (38)	NR	NR	NR	NR
**Anastasio, Fabio et al., 2021 [47]**	379	30% (113)	43% (162)	6% (23)	12% (45)	NR	NR
**Bellan, Mattia et al., 2021 [27]**	238	NR	5% (13)	3% (6)	<1% (1)	NR	NR
**Cao, Jie et al., 2021 [43]**	61	8% (5)	18% (11)	15% (9)	NR	NR	NR
**Garrigues, Eve et al. 2020 [44]**	120	55% (66)	42% (50)	17% (20)	11% (13)	NR	NR
**Han, Xaoyu et al., 2021 [28]**	114	NR	14% (16)	6% (7)	NR	NR	10% (11)
**Huang Chaolin et al., 2020 [29]**	1655	63% (1038)	26% (419)	NR	5% (75)	4% (69)	NR
**Jacobson, Kare et al., 2021 [49]**	118	31% (36)	26% (31)	8% (10)	14% (16)	3% (3)	NR
**Lerum, Tøri V. et al., 2020 [30]**	103	NR	54% (37)	NR	NR	NR	NR
**Liang, Limei et al., 2020 [31]**	76	59% (45)	61% (46)	64% (45)	62% (47)	NR	43% (33)
**Logue, Jennifer et al., 2021 [39]**	177	14% (24)	NR	NR	NR	NR	NR
**Morin, Luc et al., 2021 [36]**	478	31% (134)	16% (78)	5% (21)	8% (34)	NR	NR
**Sonnweber, Thomas et al., 2020 [32]**	133	36% (48)	NR	17% (23)	NR	NR	NR
**Sykes, Dominic et al., 2021 [50]**	134	40% (53)	60% (80)	35% (47)	18% (24)	13% (17)	NR
**Tabatabaei, Seyed M. H. et al., 2020 [33]**	52	NR	12% (6)	2% (1)	29% (15)	NR	NR
**van den Borst, B et al. 2020 [45]**	124	69% (86)	NR	NR	NR	NR	NR
**Wong, Alyson et al. 2020 [35]**	78	NR	50% (39)	23% (18)	NR	NR	NR
**Wu, Qian et al., 2021 [41]**	54	24% (13)	19% (10)	6% (3)	NR	6% (3)	NR

**Xiong, Qiutang et al., 2021 [42]**	538	28% (152)	NR	7% (38)	12% (66)	3% (17)	3% (16)
**Zhao, Yu-miao et al., 2020 [40]**	55	16% (9)	15% (8)	NR	NR	2% (1)	2% (1)

COVID-19= coronavirus disease 2019. N=population of the study. NR= not reported or reported in a different format.

Table S5. Prevalence of decreases functional capacity and health-related quality of life (HRQoL), and return to work ≥3 post COVID-19

Author	N	Decreased functional capacity % (n)	Decreased HRQoL % (n)	Return to work/no work impairment % (n)
**Baricich, Alessio et al., 2021 [48]**	204	32% (66)<br>SPPB/1-MSTST/2-MWT	NR	NR
**Bellan, Mattia et al., 2021 [27]**	238	54% (128)<br>2-minute walk/ SPPB	NR	NR
**Garrigues, Eve et al. 2020 [44]**	56	NR	NR	68% (38/56)
**Huang Chaolin et al., 2020 [29]**	1692	23% (392)<br>Below the minimum 6MWT	NR	NR
**Jacobson, Kare et al., 2021 [49]**	118	51% (54/106)<br>Any activity impairment (WPAI)	NR	61% (44/72)<br>No work impairment (WPAI)
**Liang, Limei et al., 2020 [31]**	76	NR	NR	91% (69/76)
**Logue, Jennifer et al., 2021 [39]**	166	NR	31% (51)	NR
**van den Borst, B et al. 2020 [45]**	124	20% (25)<br><80% predicted 6MWT	72% (89)<br>NCSI	NR
**Walle-Hansen, M. et al., 2021 [38]**	106	NR	54% (57)	NR
**Wong, Alyson et al. 2020 [35]**	78	NR	51% (40)	NR
**Zhao, Yu-miao et al., 2020 [40**	55	NR	NR	100% (55)

COVID-19= coronavirus disease 2019. N=population of the study. NR=not reported or reported in a different format. SPBB=short physical performance battery. NCSI= the Nijmegen Clinical Screening Instrument. 1-MSTST=1-minute sit-to-stand test. 2-MWT= 2-minute walking test. 6-MWT= 6-minture walking test. WPAI: Work Productivity and Impairment questionnaire.

Figure S1. Forest plots for follow-up chest CT (random effects model)

A. Total chest CT scan abnormalities

Study or Subgroup	Prevalence	SE	Weight	Prevalence IV, Random, 95% CI
Cao, J et al.	0.54	0.06	9.9%	0.54 [0.42, 0.66]
Han, X et al.	0.78	0.04	10.2%	0.78 [0.70, 0.86]
Huang, Ch et al..	0.53	0.03	10.4%	0.53 [0.47, 0.59]
Liang, L et al.	0.24	0.09	9.1%	0.24 [0.06, 0.42]
Shah, A et al.	0.88	0.04	10.2%	0.88 [0.80, 0.96]
Sonnweber, T et al.	0.63	0.04	10.2%	0.63 [0.55, 0.71]
Tabatabaei, S et al.	0.42	0.07	9.6%	0.42 [0.28, 0.56]
van den Borst, B et al.	0.9	0.03	10.4%	0.90 [0.84, 0.96]
Wu, Q et al.	0.2	0.05	10.1%	0.20 [0.10, 0.30]
Zhao, Y et al.	0.71	0.06	9.9%	0.71 [0.59, 0.83]
**Total (95% CI)**			**100.0%**	**0.59 [0.44, 0.73]**

Heterogeneity: Tau² = 0.05; Chi² = 245.43, df = 9 (P < 0.00001); I² = 96%
Test for overall effect: Z = 8.02 (P < 0.00001)

Prevalence IV, Random, 95% CI

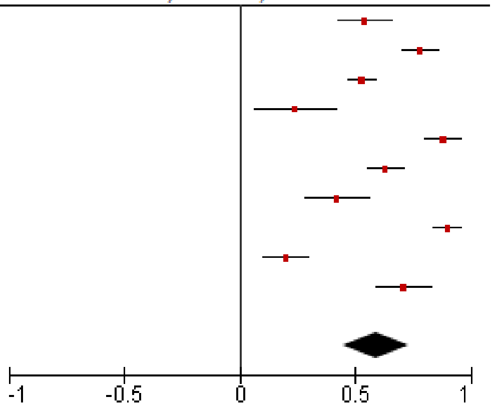

B. Ground glass opacities (GGO)

Study or Subgroup	Prevalence	SE	Weight	Prevalence IV, Random, 95% CI
Cao, J et al.	0.15	0.05	7.7%	0.15 [0.05, 0.25]
Guler, S et al.	0.58	0.07	7.4%	0.58 [0.44, 0.72]
Han, X et al.	0.21	0.04	7.8%	0.21 [0.13, 0.29]
Huang, Ch et al..	0.45	0.03	7.9%	0.45 [0.39, 0.51]
Lerum, T et al.	0.23	0.04	7.8%	0.23 [0.15, 0.31]
Liang, L et al.	0.24	0.09	7.1%	0.24 [0.06, 0.42]
Morin, L et al.	0.42	0.04	7.8%	0.42 [0.34, 0.50]
Shah, A et al.	0.83	0.05	7.7%	0.83 [0.73, 0.93]
Sonnweber, T et al.	0.39	0.04	7.8%	0.39 [0.31, 0.47]
Tabatabaei, S et al.	0.37	0.07	7.4%	0.37 [0.23, 0.51]
van den Borst, B et al.	0.87	0.04	7.8%	0.87 [0.79, 0.95]
Wu, Q et al.	0.19	0.05	7.7%	0.19 [0.09, 0.29]
Zhao, Y et al.	0.13	0.04	7.8%	0.13 [0.05, 0.21]
**Total (95% CI)**			**100.0%**	**0.39 [0.26, 0.52]**

Heterogeneity: Tau² = 0.06; Chi² = 353.66, df = 12 (P < 0.00001); I² = 97%
Test for overall effect: Z = 5.74 (P < 0.00001)

Prevalence IV, Random, 95% CI

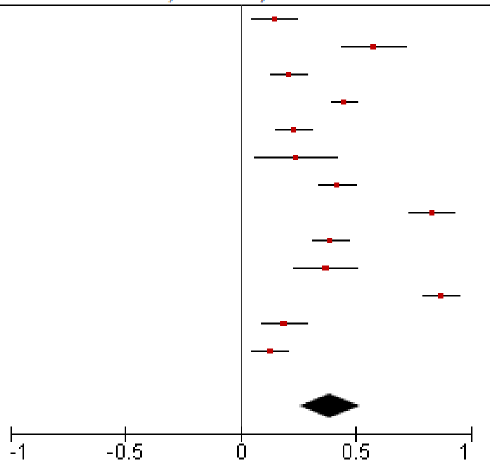

C. Interstitial thickening

Study or Subgroup	Prevalence	SE	Weight	Prevalence IV, Random, 95% CI
Cao, J et al.	0.33	0.06	16.3%	0.33 [0.21, 0.45]
Guler, S et al.	0.02	0.02	17.2%	0.02 [-0.02, 0.06]
Huang, Ch et al..	0.01	0		Not estimable
Morin, L et al.	0.19	0.03	17.1%	0.19 [0.13, 0.25]
Shah, A et al.	0.65	0.06	16.3%	0.65 [0.53, 0.77]
Sonnweber, T et al.	0.51	0.04	16.9%	0.51 [0.43, 0.59]
Zhao, Y et al.	0.27	0.06	16.3%	0.27 [0.15, 0.39]
**Total (95% CI)**			**100.0%**	**0.33 [0.13, 0.52]**

Heterogeneity: Tau² = 0.06; Chi² = 201.99, df = 5 (P < 0.00001); I² = 98%
Test for overall effect: Z = 3.28 (P = 0.001)

Prevalence IV, Random, 95% CI

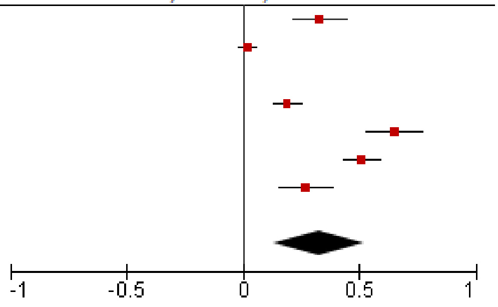

D. Parenchymal band and fibrous stripes

Study or Subgroup	Prevalence	SE	Weight	Prevalence IV, Random, 95% CI
Guler, S et al.	0.52	0.07	15.1%	0.52 [0.38, 0.66]
Huang, Ch et al..	0.16	0.02	17.7%	0.16 [0.12, 0.20]
Lerum, T et al.	0.18	0.04	16.9%	0.18 [0.10, 0.26]
Morin, L et al.	0.19	0.03	17.4%	0.19 [0.13, 0.25]
Tabatabaei, S et al.	0.19	0.05	16.4%	0.19 [0.09, 0.29]
van den Borst, B et al.	0.64	0.05	16.4%	0.64 [0.54, 0.74]
**Total (95% CI)**			**100.0%**	**0.31 [0.17, 0.44]**

Heterogeneity: Tau² = 0.03; Chi² = 100.27, df = 5 (P < 0.00001); I² = 95%
Test for overall effect: Z = 4.49 (P < 0.00001)

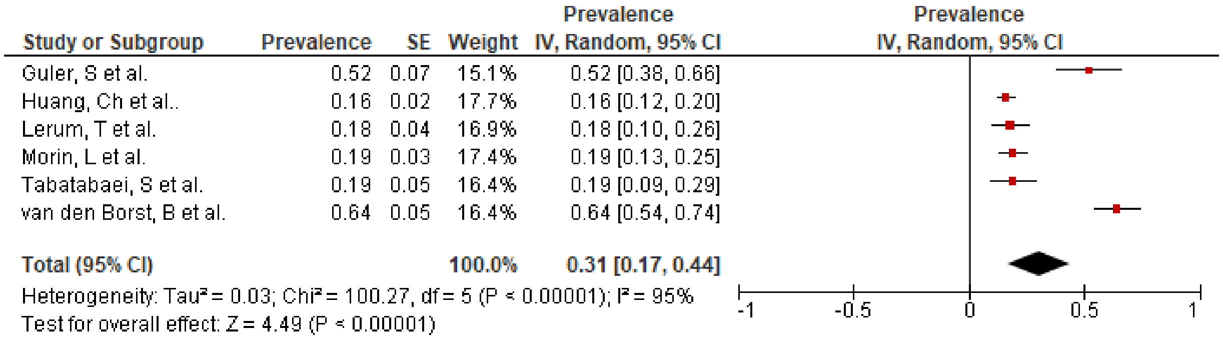

E. Bronchovascular bundle distortion or bronchiectasis

Study or Subgroup	Prevalence	SE	Weight	Prevalence IV, Random, 95% CI
Guler, S et al.	0.38	0.07	18.5%	0.38 [0.24, 0.52]
Han, X et al.	0.24	0.04	20.2%	0.24 [0.16, 0.32]
Sonnweber, T et al.	0.06	0.02	20.8%	0.06 [0.02, 0.10]
van den Borst, B et al.	0.61	0.05	19.7%	0.61 [0.51, 0.71]
Wu, Q et al.	0.02	0.02	20.8%	0.02 [-0.02, 0.06]
**Total (95% CI)**			**100.0%**	**0.26 [0.09, 0.43]**

Heterogeneity: Tau² = 0.04; Chi² = 151.91, df = 4 (P < 0.00001); I² = 97%
Test for overall effect: Z = 2.94 (P = 0.003)

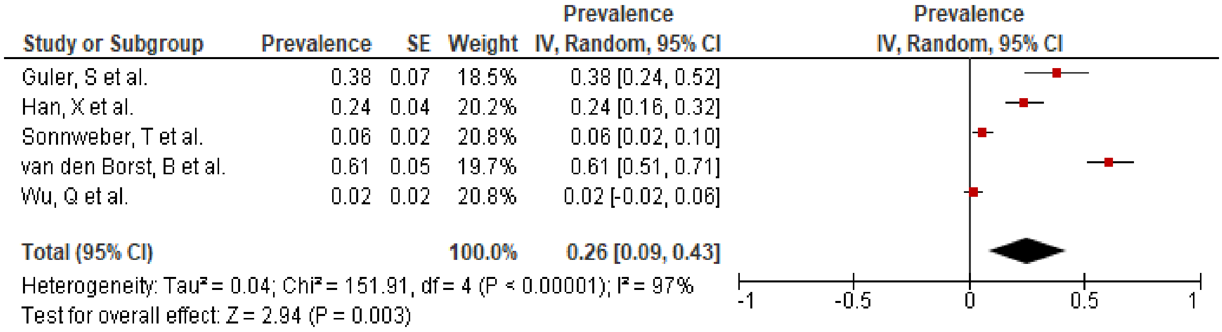

F. Pleural thickening

Study or Subgroup	Prevalence	SE	Weight	Prevalence IV, Random, 95% CI
Guler, S et al.	0.02	0.02	26.4%	0.02 [-0.02, 0.06]
Han, X et al.	0.32	0.04	23.1%	0.32 [0.24, 0.40]
Huang, Ch et al..	0.04	0.01	27.4%	0.04 [0.02, 0.06]
Wu, Q et al.	0.09	0.04	23.1%	0.09 [0.01, 0.17]
**Total (95% CI)**			**100.0%**	**0.11 [0.02, 0.20]**

Heterogeneity: Tau² = 0.01; Chi² = 49.79, df = 3 (P < 0.00001); I² = 94%
Test for overall effect: Z = 2.37 (P = 0.02)

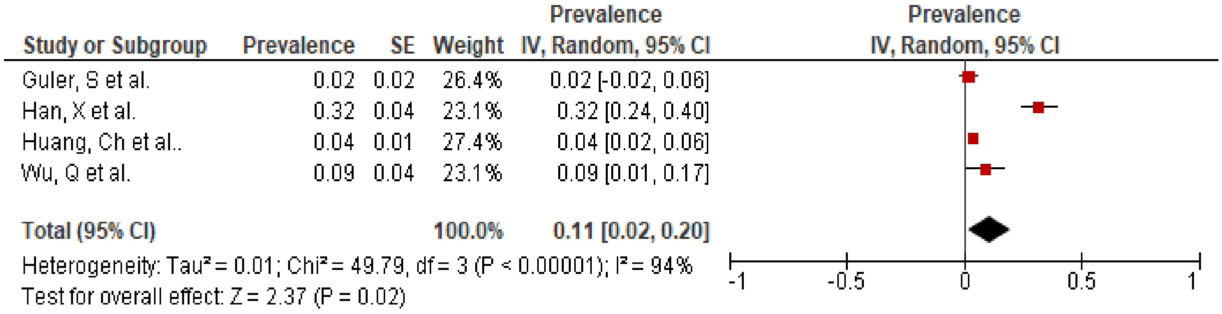

G. Consolidations

Study or Subgroup	Prevalence	SE	Weight	Prevalence IV, Random, 95% CI
Guler, S et al.	0.29	0.06	10.7%	0.29 [0.17, 0.41]
Han, X et al.	0.03	0.01	31.2%	0.03 [0.01, 0.05]
Huang, Ch et al..	0.01	0.01	31.2%	0.01 [-0.01, 0.03]
Sonnweber, T et al.	0.07	0.02	26.8%	0.07 [0.03, 0.11]
**Total (95% CI)**			**100.0%**	**0.06 [0.02, 0.11]**

Heterogeneity: Tau² = 0.00; Chi² = 26.74, df = 3 (P < 0.00001); I² = 89%
Test for overall effect: Z = 2.61 (P = 0.009)

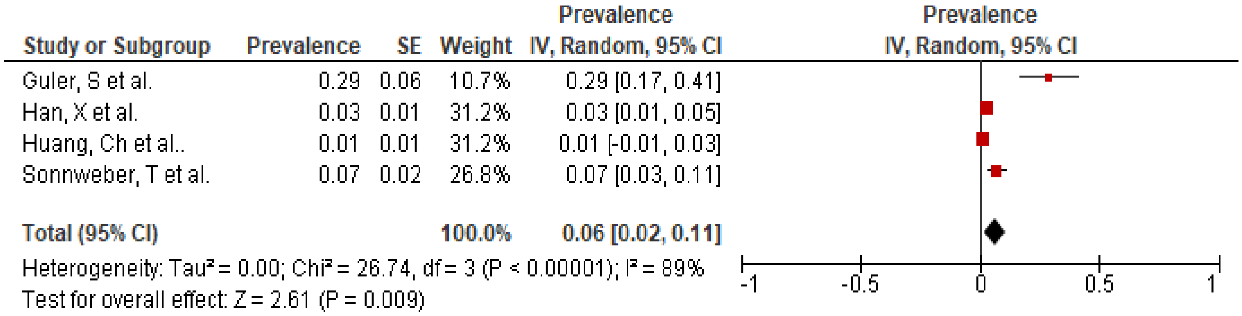

Figure S2. Forest plots for follow-up pulmonary function (random effects model)

A. Abnormal pulmonary function test (PFT)

Study or Subgroup	Prevalence	SE	Weight	Prevalence IV, Random, 95% CI
Cao, J et al.	0.1	0.04	14.7%	0.10 [0.02, 0.18]
Liang, L et al.	0.42	0.06	14.1%	0.42 [0.30, 0.54]
Morin, L et al.	0.63	0.04	14.7%	0.63 [0.55, 0.71]
Shah, A et al.	0.58	0.06	14.1%	0.58 [0.46, 0.70]
Sonnweber, T et al.	0.36	0.04	14.7%	0.36 [0.28, 0.44]
Wu, Q et al.	0.41	0.07	13.7%	0.41 [0.27, 0.55]
Zhao, Y et al.	0.25	0.06	14.1%	0.25 [0.13, 0.37]
**Total (95% CI)**			**100.0%**	**0.39 [0.24, 0.55]**

Heterogeneity: Tau² = 0.04; Chi² = 104.74, df = 6 (P < 0.00001); I² = 94%
Test for overall effect: Z = 4.96 (P < 0.00001)

Prevalence IV, Random, 95% CI

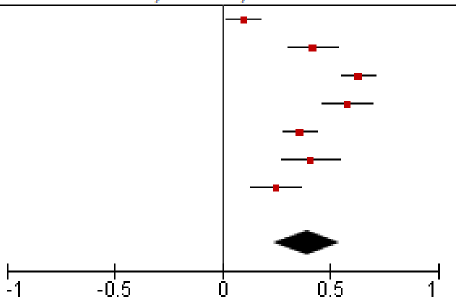

B. Diffusion capacity

Study or Subgroup	Prevalence	SE	Weight	Prevalence IV, Random, 95% CI
Abdallah, S et al.	0.46	0.06	7.6%	0.46 [0.34, 0.58]
Bella, M et al.	0.52	0.03	9.0%	0.52 [0.46, 0.58]
Han, X et al.	0.26	0.04	8.6%	0.26 [0.18, 0.34]
Huang, Ch et al..	0.33	0.03	9.0%	0.33 [0.27, 0.39]
Lerum, T et al.	0.23	0.04	8.6%	0.23 [0.15, 0.31]
Liang, L et al.	0.2	0.05	8.1%	0.20 [0.10, 0.30]
Morin, L et al.	0.22	0.03	9.0%	0.22 [0.16, 0.28]
Shah, A et al.	0.52	0.06	7.6%	0.52 [0.40, 0.64]
Sonnweber, T et al.	0.21	0.04	8.6%	0.21 [0.13, 0.29]
van den Borst, B et al.	0.34	0.04	8.6%	0.34 [0.26, 0.42]
Wu, Q et al.	0.31	0.06	7.6%	0.31 [0.19, 0.43]
Zhao, Y et al.	0.16	0.05	8.1%	0.16 [0.06, 0.26]
**Total (95% CI)**			**100.0%**	**0.31 [0.24, 0.38]**

Heterogeneity: Tau² = 0.01; Chi² = 103.06, df = 11 (P < 0.00001); I² = 89%
Test for overall effect: Z = 8.60 (P < 0.00001)

Prevalence IV, Random, 95% CI

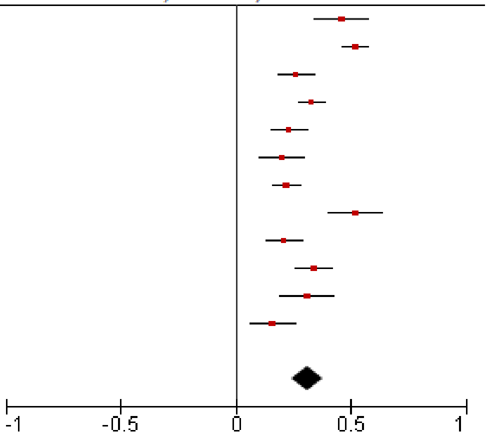

C. Restrictive pattern

Study or Subgroup	Prevalence	SE	Weight	Prevalence IV, Random, 95% CI
Abdallah, S et al.	0.11	0.04	11.5%	0.11 [0.03, 0.19]
Huang, Ch et al..	0.16	0.02	14.8%	0.16 [0.12, 0.20]
Lerum, T et al.	0.07	0.02	14.8%	0.07 [0.03, 0.11]
Shah, A et al.	0.23	0.05	9.8%	0.23 [0.13, 0.33]
Sonnweber, T et al.	0.26	0.04	11.5%	0.26 [0.18, 0.34]
van den Borst, B et al.	0.06	0.02	14.8%	0.06 [0.02, 0.10]
Wu, Q et al.	0.07	0.04	11.5%	0.07 [-0.01, 0.15]
Zhao, Y et al.	0.07	0.04	11.5%	0.07 [-0.01, 0.15]
**Total (95% CI)**			**100.0%**	**0.12 [0.08, 0.17]**

Heterogeneity: Tau² = 0.00; Chi² = 38.32, df = 7 (P < 0.00001); I² = 82%
Test for overall effect: Z = 5.02 (P < 0.00001)

Prevalence IV, Random, 95% CI

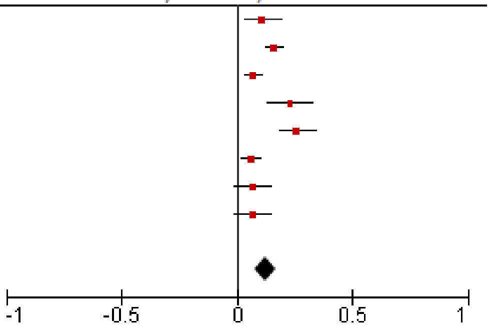

D. Obstructive pattern

Study or Subgroup	Prevalence	SE	Weight	Prevalence IV, Random, 95% CI
Abdallah, S et al.	0.11	0.04	4.6%	0.11 [0.03, 0.19]
Huang, Ch et al..	0.06	0.01	52.9%	0.06 [0.04, 0.08]
Liang, L et al.	0.07	0.03	8.1%	0.07 [0.01, 0.13]
Shah, A et al.	0.12	0.04	4.6%	0.12 [0.04, 0.20]
Sonnweber, T et al.	0.08	0.02	17.1%	0.08 [0.04, 0.12]
van den Borst, B et al.	0.11	0.03	8.1%	0.11 [0.05, 0.17]
Zhao, Y et al.	0.11	0.04	4.6%	0.11 [0.03, 0.19]
**Total (95% CI)**			**100.0%**	**0.08 [0.06, 0.09]**

Heterogeneity: $Tau^2 = 0.00$; $Chi^2 = 6.44$, $df = 6$ ($P = 0.38$); $I^2 = 7\%$
Test for overall effect: $Z = 8.68$ ($P < 0.00001$)

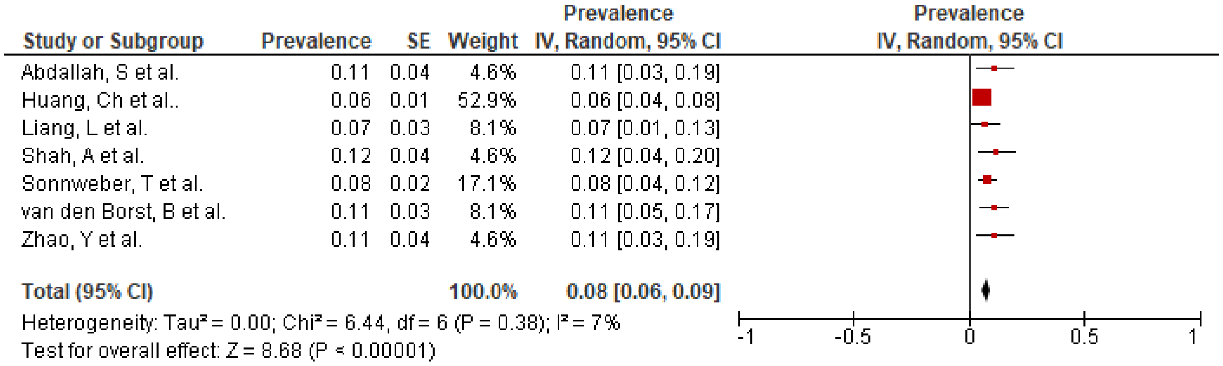

Figure S3. Forest plots for follow-up respiratory symptoms (random effects model)

A. Fatigue

Study or Subgroup	Prevalence	SE	Weight	Prevalence IV, Random, 95% CI
Abdallah, S et al.	0.71	0.06	6.4%	0.71 [0.59, 0.83]
Anastasio, F et al.	0.3	0.02	6.9%	0.30 [0.26, 0.34]
Cao, J et al.	0.08	0.04	6.7%	0.08 [0.00, 0.16]
Garrigues, E et al.	0.55	0.05	6.6%	0.55 [0.45, 0.65]
Huang, Ch et al..	0.63	0.01	6.9%	0.63 [0.61, 0.65]
Jacobson, K et al.	0.31	0.04	6.7%	0.31 [0.23, 0.39]
Liang, L et al.	0.59	0.06	6.4%	0.59 [0.47, 0.71]
Logue, J et al.	0.14	0.03	6.8%	0.14 [0.08, 0.20]
Morin, L et al.	0.31	0.02	6.9%	0.31 [0.27, 0.35]
Sonnweber, T et al.	0.36	0.04	6.7%	0.36 [0.28, 0.44]
Sykes, D et al.	0.4	0.04	6.7%	0.40 [0.32, 0.48]
van den Borst, B et al.	0.69	0.04	6.7%	0.69 [0.61, 0.77]
Wu, Q et al.	0.24	0.06	6.4%	0.24 [0.12, 0.36]
Xiong, Q et al.	0.28	0.02	6.9%	0.28 [0.24, 0.32]
Zhao, Y et al.	0.16	0.05	6.6%	0.16 [0.06, 0.26]
**Total (95% CI)**			**100.0%**	**0.38 [0.27, 0.49]**

Heterogeneity: Tau² = 0.04; Chi² = 822.80, df = 14 (P < 0.00001); I² = 98%
Test for overall effect: Z = 6.88 (P < 0.00001)

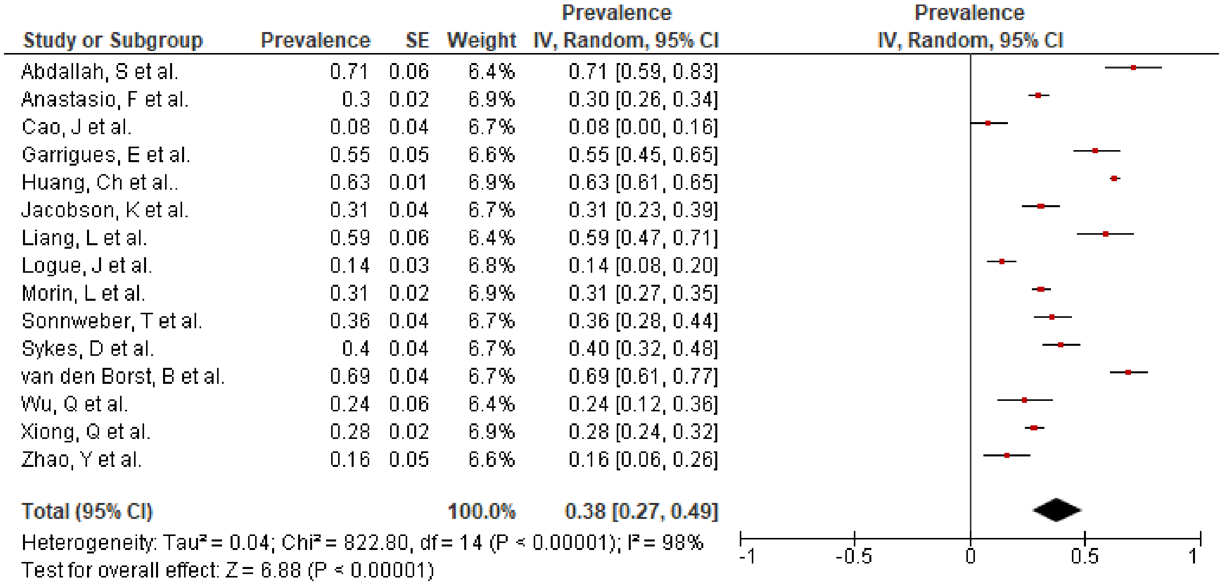

B. Dyspnea

Study or Subgroup	Prevalence	SE	Weight	Prevalence IV, Random, 95% CI
Abdallah, S et al.	0.6	0.06	5.9%	0.60 [0.48, 0.72]
Anastasio, F et al.	0.43	0.03	6.5%	0.43 [0.37, 0.49]
Bella, M et al.	0.05	0.01	6.7%	0.05 [0.03, 0.07]
Cao, J et al.	0.18	0.05	6.1%	0.18 [0.08, 0.28]
Garrigues, E et al.	0.42	0.05	6.1%	0.42 [0.32, 0.52]
Han, X et al.	0.14	0.03	6.5%	0.14 [0.08, 0.20]
Huang, Ch et al..	0.26	0.01	6.7%	0.26 [0.24, 0.28]
Jacobson, K et al.	0.26	0.04	6.3%	0.26 [0.18, 0.34]
Lerum, T et al.	0.54	0.06	5.9%	0.54 [0.42, 0.66]
Liang, L et al.	0.61	0.06	5.9%	0.61 [0.49, 0.73]
Morin, L et al.	0.16	0.02	6.6%	0.16 [0.12, 0.20]
Sykes, D et al.	0.6	0.04	6.3%	0.60 [0.52, 0.68]
Tabatabaei, S et al.	0.12	0.04	6.3%	0.12 [0.04, 0.20]
Wong, A et al.	0.5	0.06	5.9%	0.50 [0.38, 0.62]
Wu, Q et al.	0.19	0.05	6.1%	0.19 [0.09, 0.29]
Zhao, Y et al.	0.15	0.05	6.1%	0.15 [0.05, 0.25]
**Total (95% CI)**			**100.0%**	**0.32 [0.24, 0.40]**

Heterogeneity: Tau² = 0.03; Chi² = 602.39, df = 15 (P < 0.00001); I² = 98%
Test for overall effect: Z = 7.60 (P < 0.00001)

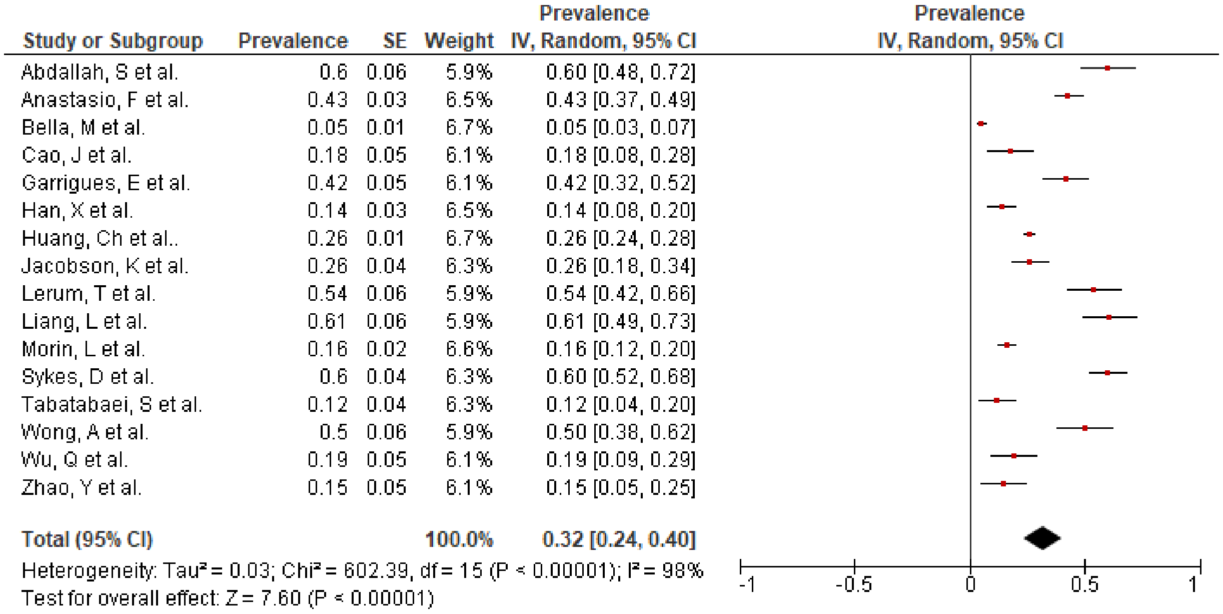

C. Chest Paint/tightness

Study or Subgroup	Prevalence	SE	Weight	Prevalence IV, Random, 95% CI
Anastasio, F et al.	0.12	0.02	12.3%	0.12 [0.08, 0.16]
Bella, M et al.	0.01	0		Not estimable
Garrigues, E et al.	0.11	0.03	11.2%	0.11 [0.05, 0.17]
Huang, Ch et al..	0.05	0.01	13.2%	0.05 [0.03, 0.07]
Jacobson, K et al.	0.14	0.03	11.2%	0.14 [0.08, 0.20]
Liang, L et al.	0.62	0.06	7.3%	0.62 [0.50, 0.74]
Morin, L et al.	0.08	0.01	13.2%	0.08 [0.06, 0.10]
Sykes, D et al.	0.18	0.03	11.2%	0.18 [0.12, 0.24]
Tabatabaei, S et al.	0.29	0.06	7.3%	0.29 [0.17, 0.41]
Xiong, Q et al.	0.12	0.01	13.2%	0.12 [0.10, 0.14]
**Total (95% CI)**			**100.0%**	**0.16 [0.12, 0.21]**

Heterogeneity: Tau² = 0.00; Chi² = 127.82, df = 8 (P < 0.00001); I² = 94%
Test for overall effect: Z = 6.75 (P < 0.00001)

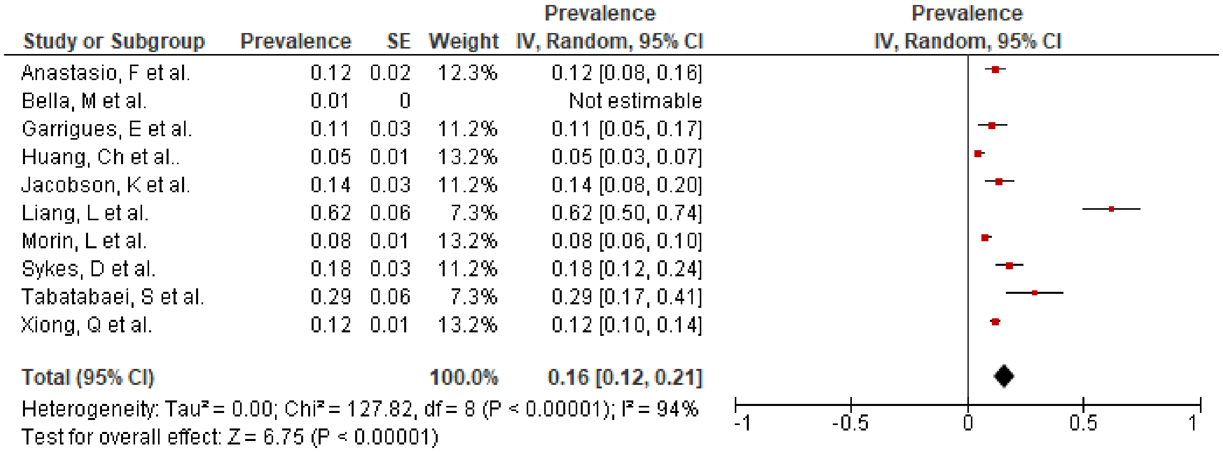

D. Cough

Study or Subgroup	Prevalence	SE	Weight	Prevalence IV, Random, 95% CI
Anastasio, F et al.	0.06	0.01	8.4%	0.06 [0.04, 0.08]
Bella, M et al.	0.03	0.01	8.4%	0.03 [0.01, 0.05]
Cao, J et al.	0.15	0.05	5.5%	0.15 [0.05, 0.25]
Garrigues, E et al.	0.17	0.03	7.1%	0.17 [0.11, 0.23]
Han, X et al.	0.06	0.02	7.9%	0.06 [0.02, 0.10]
Jacobson, K et al.	0.08	0.03	7.1%	0.08 [0.02, 0.14]
Liang, L et al.	0.64	0.06	4.7%	0.64 [0.52, 0.76]
Morin, L et al.	0.05	0.01	8.4%	0.05 [0.03, 0.07]
Sonnweber, T et al.	0.17	0.03	7.1%	0.17 [0.11, 0.23]
Sykes, D et al.	0.35	0.04	6.3%	0.35 [0.27, 0.43]
Tabatabaei, S et al.	0.02	0.02	7.9%	0.02 [-0.02, 0.06]
Wong, A et al.	0.23	0.05	5.5%	0.23 [0.13, 0.33]
Wu, Q et al.	0.06	0.03	7.1%	0.06 [0.00, 0.12]
Xiong, Q et al.	0.07	0.01	8.4%	0.07 [0.05, 0.09]
**Total (95% CI)**			**100.0%**	**0.13 [0.09, 0.17]**

Heterogeneity: Tau² = 0.00; Chi² = 201.22, df = 13 (P < 0.00001); I² = 94%
Test for overall effect: Z = 6.77 (P < 0.00001)

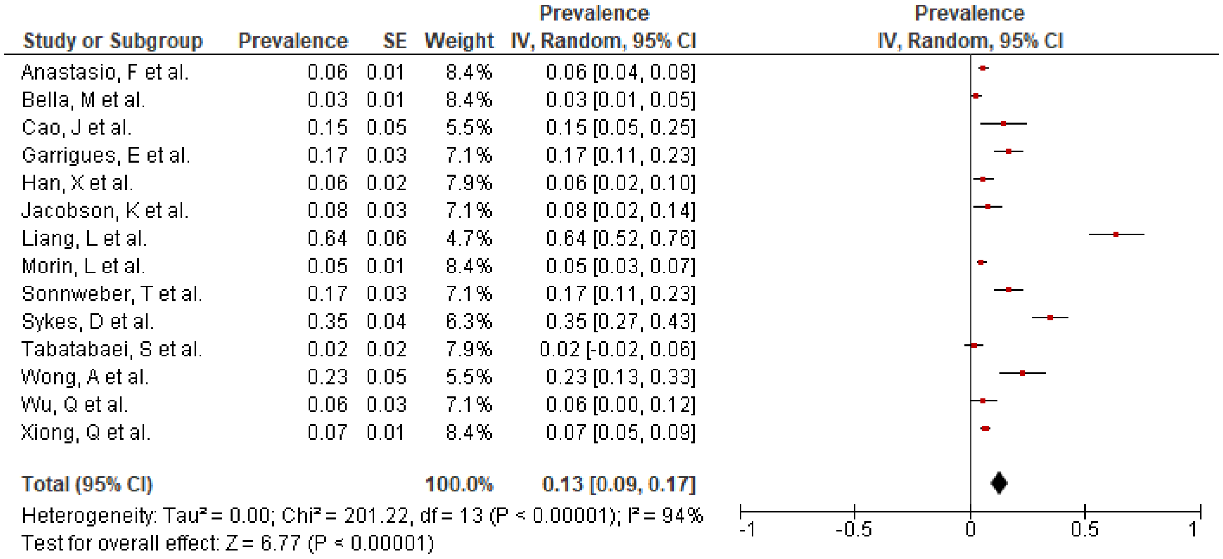

E. Sputum

Study or Subgroup	Prevalence	SE	Weight	Prevalence IV, Random, 95% CI
Han, X et al.	0.1	0.03	25.5%	0.10 [0.04, 0.16]
Liang, L et al.	0.43	0.06	19.2%	0.43 [0.31, 0.55]
Xiong, Q et al.	0.03	0.01	28.2%	0.03 [0.01, 0.05]
Zhao, Y et al.	0.02	0.02	27.1%	0.02 [-0.02, 0.06]
**Total (95% CI)**			**100.0%**	**0.12 [0.03, 0.21]**

Heterogeneity: Tau² = 0.01; Chi² = 48.20, df = 3 (P < 0.00001); I² = 94%
Test for overall effect: Z = 2.66 (P = 0.008)

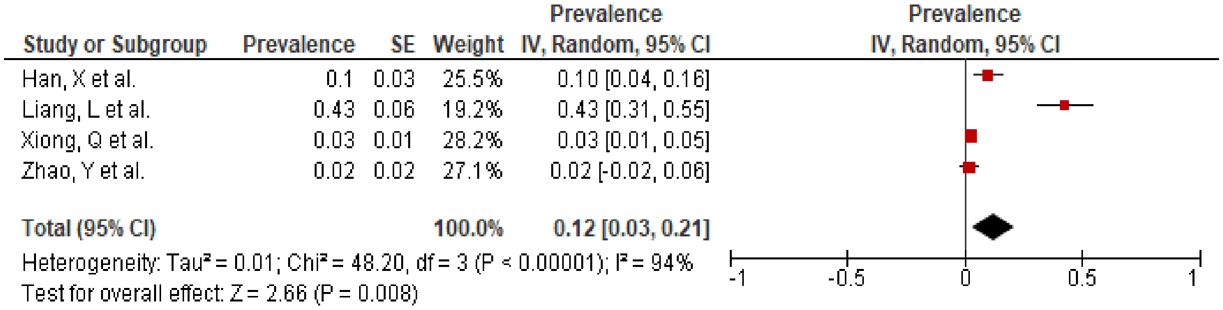

F. Sore throat

Study or Subgroup	Prevalence	SE	Weight	Prevalence IV, Random, 95% CI
Huang, Ch et al..	0.04	0		Not estimable
Jacobson, K et al.	0.03	0.01	28.6%	0.03 [0.01, 0.05]
Sykes, D et al.	0.13	0.03	12.0%	0.13 [0.07, 0.19]
Wu, Q et al.	0.06	0.03	12.0%	0.06 [0.00, 0.12]
Xiong, Q et al.	0.03	0.01	28.6%	0.03 [0.01, 0.05]
Zhao, Y et al.	0.02	0.02	18.8%	0.02 [-0.02, 0.06]
**Total (95% CI)**			**100.0%**	**0.04 [0.02, 0.07]**

Heterogeneity: $Tau^2 = 0.00$; $Chi^2 = 11.78$, df = 4 (P = 0.02); $I^2 = 66\%$
Test for overall effect: Z = 3.40 (P = 0.0007)

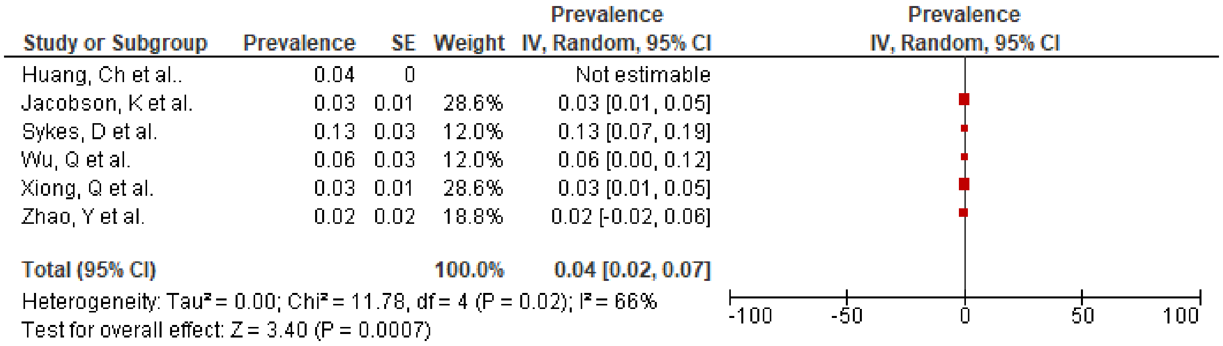

Figure S4. Functional capacity and Health-related quality of life (HRQoL)

A. Functional capacity (performance-based tests)

Study or Subgroup	Prevalence	SE	Weight	Prevalence IV, Random, 95% CI
Baricich, A et al.	0.32	0.03	20.2%	0.32 [0.26, 0.38]
Bella, M et al.	0.54	0.03	20.2%	0.54 [0.48, 0.60]
Huang, Ch et al..	0.23	0.01	21.0%	0.23 [0.21, 0.25]
Jacobson, K et al.	0.51	0.05	18.9%	0.51 [0.41, 0.61]
van den Borst, B et al.	0.2	0.04	19.6%	0.20 [0.12, 0.28]
**Total (95% CI)**			**100.0%**	**0.36 [0.22, 0.49]**

Heterogeneity: $Tau^2 = 0.02$; $Chi^2 = 125.88$, df = 4 (P < 0.00001); $I^2 = 97\%$
Test for overall effect: Z = 5.23 (P < 0.00001)

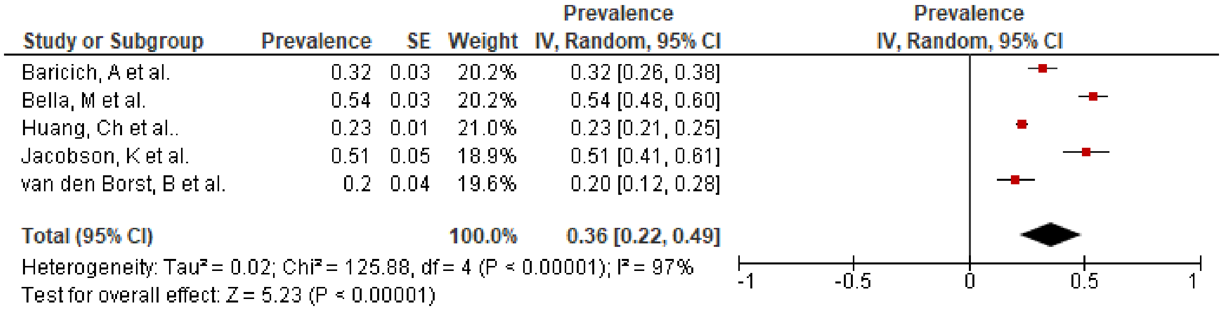

B. HRQoL

Study or Subgroup	Prevalence	SE	Weight	Prevalence IV, Random, 95% CI
Logue, J et al.	0.31	0.04	25.5%	0.31 [0.23, 0.39]
van den Borst, B et al.	0.72	0.04	25.5%	0.72 [0.64, 0.80]
Walle-Hansen, M et al.	0.54	0.05	24.9%	0.54 [0.44, 0.64]
Wong, A et al.	0.51	0.06	24.2%	0.51 [0.39, 0.63]
**Total (95% CI)**			**100.0%**	**0.52 [0.33, 0.71]**

Heterogeneity: $Tau^2 = 0.04$; $Chi^2 = 52.75$, df = 3 (P < 0.00001); $I^2 = 94\%$
Test for overall effect: Z = 5.35 (P < 0.00001)

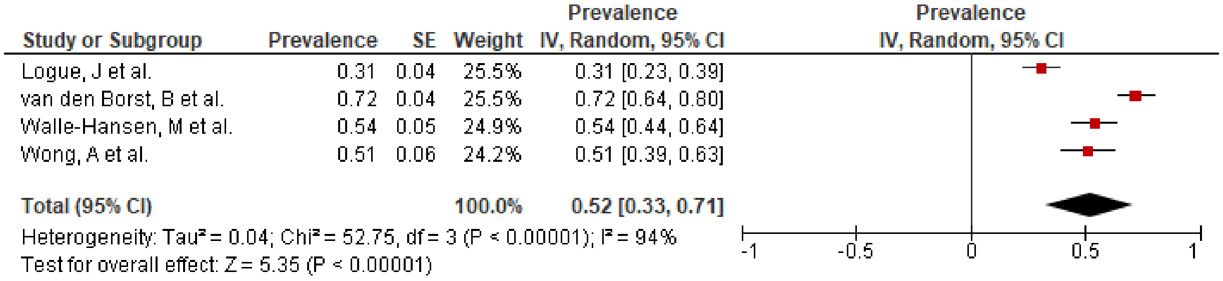

Supplement: Supplementary file 1 [file biomedicines-09-00900-s001.zip › biomedicines-1311136-supplementary.pdf]
